# Characterization of Main Responsive Genes Reveals Their Regulatory Network Attended by Multi-Biological Metabolic Pathways in Paclobutrazol (PAC)-Modulated Grape Seed Development (GSD) at the Stone-Hardening Stage

**DOI:** 10.3390/ijms26031102

**Published:** 2025-01-27

**Authors:** Rana Badar Aziz, Ji Wei, Qiqi Wu, Siyan Song, Hui Yang, Xinpeng Chen, Ying Wang, Ruiqiang Chao, Naila Mir Baz, Haitao Chen, Yuxuan Song, Jinggui Fang, Chen Wang

**Affiliations:** 1 College of Horticulture, Nanjing Agricultural University, Nanjing 210095, China; 2022104339@stu.njau.edu.cn (R.B.A.); w17861701220@163.com (Q.W.); 2012088@njau.edu.cn (S.S.); 2023804248@stu.njau.edu.cn (H.Y.); 2023104032@stu.njau.edu.cn (X.C.); 2023104031@stu.njau.edu.cn (Y.W.); chaoruiqiang1026@163.com (R.C.); chenhaitao@stu.njau.edu.cn (H.C.); 15149149427@163.com (Y.S.); fangjj1215@163.com (J.F.); 2 College of Horticulture, Shanxi Agricultural University, Taigu 030031, China; sxswrcb@163.com; 3 College of Horticulture, Hebei Agricultural University, Baoding 071000, China; nailamirbazbarcha@gmail.com

**Keywords:** paclobutrazol, *Vitis vinifera*, grape seed development, phytohormones, multi-biological metabolic pathways, regulatory network

## Abstract

Paclobutrazol (PAC) is a significant inhibitor of gibberellin biosynthesis that profoundly influences grape seed development (GSD) through the modulation of key molecular pathways. Here, we identified 6659 differentially expressed genes (DEGs) in GSD under PAC treatment, with 3601 up-regulated and 3058 down-regulated. An analysis of hormone-associated DEGs revealed that auxin-related genes (16) were the most up-regulated, followed by genes associated with brassinosteroid and ABA. In contrast, cytokinin- and gibberellin-related genes exhibited a suppressive response. PAC treatment also triggered extensive reprogramming of metabolic pathways, including 44 genes involved in starch and sucrose metabolism (24 up-regulated, 20 down-regulated), 101 cell wall-related genes (53 up-regulated, 48 down-regulated), and 110 transcription factors (77 up-regulated, 33 down-regulated). A cis-element analysis of the promoters of 76 hormone-responsive genes identified 14 types of hormone-responsive cis-elements, with ABRE being the most prevalent. Genes responsible for inactivating active hormones, such as ABA-*VvPP2CA, IAA-VvGH3.1*, and *CK-VvARR9-1*, were also identified. Concurrently, PAC negatively regulated hormone-active genes, including *BR-VvXTH25*, *SA-VvTGA21-3*, and *JA-VvTIFY3B*, leading to reduced levels of these hormones. PAC modulates GSD by mediating the dynamic balance of multi-hormone accumulations. Furthermore, development-related cis-elements such as the AACA-motif, AAGAA-motif, AC-I, AC-II, O2-site, as-1, CAT-box, CCAAT-box, circadian, GCN4-motif, RY-element, HD-Zip 1, HD-Zip 3, MSA-like, MYB-like sequence, MYB-binding site, and MYB recognition site, were found in key DEGs involved in starch and sucrose metabolism, cell wall remodeling, and epigenetic regulation. This indicates that these pathways are responsive to PAC modulation during GSD. Finally, we developed a comprehensive regulatory network to illustrate the PAC-mediated pathways involved in GSD. This network integrates multi-hormonal signaling, cell wall remodeling, epigenetic regulation, and transcription factors, highlighting PAC’s pivotal role in GSD. Our findings provide new insights into the complex mechanisms underlying PAC’s effects on grapevine development.

## 1. Introduction

Grapes (*Vitis vinifera*) are among the most important perennial fruit crops, widely cultivated worldwide, and are celebrated for their versatility in winemaking, culinary applications, and nutritional benefits [1]. Seeds play a crucial role in grape berry growth and development, functioning as the central tissues responsible for GA production. Paclobutrazol (PAC), a synthetic plant growth retardant, is a key inhibitor of endogenous GA synthesis that has been increasingly utilized to modulate plant growth and development, particularly in the grape industry [2]. The development of grape seeds (GSD) is a complex interplay of genetic predispositions and environmental factors, which intricately regulates the growth and maturation of various grapevine organs, especially the seeds [3,4]. Understanding how GSD is modulated has significant implications for fruit quality and yield, prompting research into various approaches, including the application of plant growth regulators (PGRs) like PAC. However, by inhibiting gibberellin biosynthesis, PAC interferes with several growth processes, such as cell elongation, seed germination, and fruit development [5]. Lower GA levels due to PAC application lead to decreased cellular expansion in berry tissues, ultimately affecting berry size and shape [6,7]. Recent studies indicated that PAC influences the induction of parthenocarpic fruit development, where fruits form without fertilization [8]. PAC’s role in inhibiting GA-induced fruit growth highlights its impact on fruit set and subsequent fruit development [9]. By modulating GA biosynthesis, PAC affects key processes associated with cell division and expansion, thereby regulating fruit size and development. This inhibitory effect on GA pathways suggests that PAC is crucial for maintaining the hormonal balance necessary for successful fruit formation and growth, particularly in parthenocarpy systems [10,11].

While previous research has examined the effects of PAC in grape growth and development [12], its role as an exogenous growth regulator in seed development and related networks remains unexplored. Seed formation involves the establishment of hormonal gradients through multiple auxin biosynthesis pathways, with diverse auxins exerting unique effects [13,14]. Given that PAC is a synthesized growth regulator that inhibits GA synthesis, its application plays a significant role in GSD by mediating various biological metabolic pathways, including multiple hormonal signals. This work aims to investigate the effects of PAC application on grape seeds, focusing on elucidating PAC-mediated biological metabolic pathways.

## 2. Result

### 2.1. Screen the Main Responsive Genes Involved in Various Pathways in PAC-Mediated GSD

**Figure 1:**
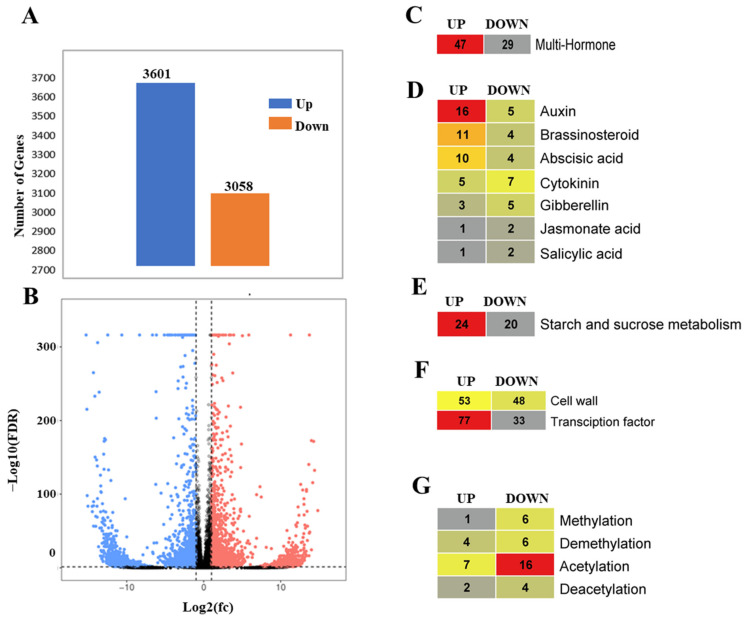
(**A**) Significant up-regulated (blue) and down-regulated (orange) genes during GSD under PAC treatment. (**B**) Volcano plot illustrating gene expression changes based on |log2 fold change| and *p*-value during PAC treatment in GSD. (**C**) Overview of hormone-responsive genes in GSD with total gene counts under PAC. (**D**) Breakdown of multi-hormone-related gene counts by regulation type. (**E**) Starch and sucrose metabolism-related genes under PAC treatment. (**F**) Cell wall, transcription factor (TF) genes in GSD. (**G**) Epigenetic regulation-related gene counts in GSD under PAC treatment.

In the analysis of GSD under PAC treatment, 34,593 mRNAs were examined, resulting in the identification of 6659 DEGs. Among these, 3601 genes were significantly up-regulated (|log2FC| > 1, FDR < 0.05), while 3058 genes showed significant down-regulation, indicating reduced expression. This analysis is visually represented in a volcano plot, which highlights the dynamic impact of PAC on the gene expression landscape during GSD, distinctly illustrating the differences between up-regulated and down-regulated genes (Figure 1A,B).

Further analysis of the primary response pathways derived from the DEGs revealed 76 genes associated with various hormones. Of these, 47 genes were up-regulated, indicating increased activity, whereas 29 genes were down-regulated, suggesting a decrease in activity in response to the treatment (Figure 1C).

### 2.2. Identification of the Main Responsive Genes Involved in Multi-Hormone Pathways in PAC-Mediated GSD

Since PAC inhibits GA biosynthesis, we identified several hormone-related genes affected by this inhibition. Consequently, we uncovered a variety of genes that regulate GSD in response to PAC treatment. A total of 76 hormone-related genes were identified. Auxin displayed the highest number of up-regulated genes, with 16 genes up-regulated and five down-regulated, highlighting its critical role in signaling. Brassinosteroid also showed strong activation, characterized by 11 up-regulated genes and four down-regulated genes. Abscisic acid (ABA) was significantly involved with 10 up-regulated and four down-regulated genes. In contrast, cytokinin exhibited a more suppressive pattern, with five genes up-regulated and seven down-regulated, suggesting an inhibition of cytokinin signaling. Gibberellin showed an expression pattern with three genes up-regulated and five down-regulated. Jasmonic acid had one up-regulated and two down-regulated genes, while salicylic acid also showed one up-regulated and two down-regulated genes (Supplementary Sheet S1 and Figure 1D).

### 2.3. Characterization of the Main Responsive Genes Involved in the Multi-Hormone in PAC-Mediated GSD

**Figure 2:**
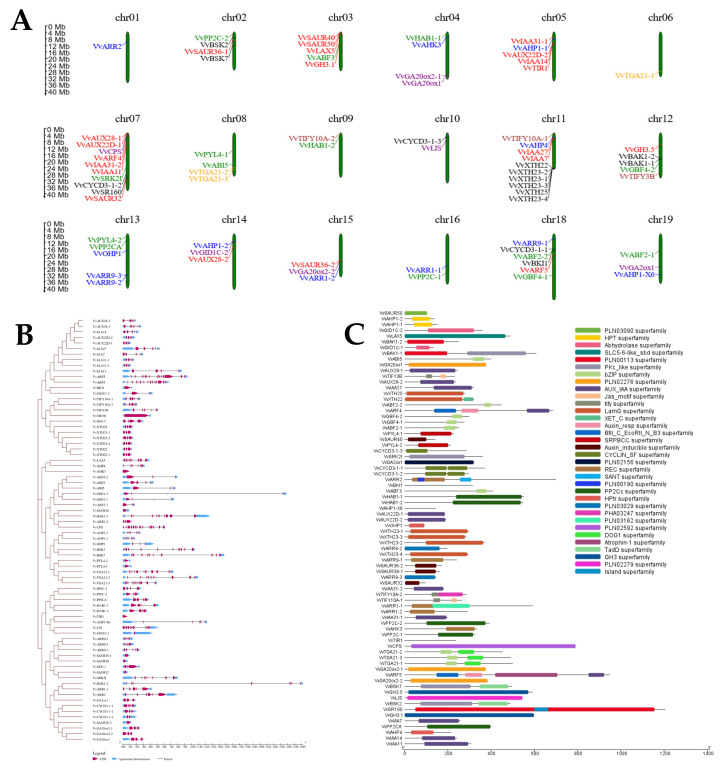
Genomic and functional analysis of hormone-responsive genes in GSD: (**A**) Chromosomal locations of hormone genes: Auxin (red), CK (blue), BR (black), ABA (green), JA (brown), SA (orange), and GA (purple). (**B**) Phylogenetic tree illustrating evolutionary relationships among hormone-related genes alongside gene structure analysis showing their exon-intron arrangements. (**C**) Conserved domain analysis highlighting key functional motifs across hormone gene families.

The distribution of 76 multi-hormone-related genes across the grapevine genome exhibited significant variability. These genes were localized to 18 of the 19 chromosomes, indicating an uneven distribution pattern. Chromosomes 7 and 11 were prominent, each hosting 10 genes. Chromosome 18 closely followed with six genes, while chromosomes 3, 5, 12, and 13 contained five genes. The remaining chromosomes (1, 2, 4, 6, 8, 9, 10, 14, 15, 16, and 19) carried between one and four genes. Interestingly, one gene lacked a chromosome assignment, highlighting an incomplete map of chromosomal localization. This uneven distribution suggests that specific chromosomes may play a more significant role in regulating hormone-related processes in GSD (Figure 2A).

Phylogenetic analysis revealed distinct classifications for proteins encoded by genes associated with multi-hormone regulation. All hormone-responsive genes clustered together, indicating their evolutionary relationship; however, notable diversity was observed in the number of exons and introns present across these genes. Specifically, eleven genes—*VvBKI1, VvAHK3, VvSAUR40, VvPYL4-2, VvPYL4-1, VvTIRI, VvGIDIC-1*, *VvSAUR36-1, VvSAUR50, VvSAUR32, and VvSAUR32-2*—were identified as intronless in the grape genome, suggesting a streamlined genomic architecture for these particular genes. Conversely, *VvGBF4-2* and *VvBAK1-2* were characterized as the most extended genes within the hormone-responsive genes family, containing eight and four exons, respectively, which may indicate their complex functional roles. Additionally, three genes, *VvARF5, VvARF4*, and *VvCPS*, each exhibited a high exon count of 14, reflecting potential regulatory complexity. The remaining genes displayed a wide range of exon numbers, further underscoring the variability in gene structure (Figure 2B).

Conserved domain analysis revealed that 34 superfamily domains were identified among the 76 hormone-responsive genes analyzed. *VvARR5* contained four superfamily domains, while *VvARR4* and *VvARR2* each possessed three. Other proteins contained one or two superfamily domains, with proteins of similar functions generally sharing the same domains. Interestingly, *VvBKI1, VvAHPI-X6*, and *VvTIR1* were found to lack superfamily domains (Figure 2C).

### 2.4. Identification of Starch and Sucrose Metabolism, Cell Wall Genes, Epigenetic Modifications, and Transcription Factors Regulated by Hormone-Responsive Genes in GSD

The influence of hormone-responsive genes in the presence of PAC during GSD revealed a complex regulatory network involving various metabolic pathways and gene expressions. PAC, an inhibitor of gibberellin (GA) biosynthesis, likely alters the synthesis and accumulation of other hormones, including abscisic acid (ABA), cytokinins (CKs), and auxins, thereby affecting key processes such as cell growth, development, and epigenetic regulation. In the starch and sucrose metabolism pathway, 44 genes were identified, with 24 up-regulated and 20 down-regulated, demonstrating differential responses to PAC treatment (Figure 1E). These changes suggest that PAC mediates the synthesis and accumulation of multiple hormones by regulating related gene expression levels during GSD. Similarly, 101 cell wall-related genes were identified, with 53 genes up-regulated and 48 down-regulated, indicating the significant modulation of cell wall remodeling. This regulation may be linked to alterations in hormonal balance, especially the accumulation of hormones that affect cell expansion and structural integrity.

Transcription factors exhibited notable changes, with 110 genes identified, 77 up-regulated, and 33 down-regulated, highlighting the dynamic role of transcriptional regulation under PAC influence (Figure 1F). The altered hormonal landscape likely influences these changes. Furthermore, hormone-responsive gene interactions impacted epigenetic modifications. Seven genes related to methylation were identified, with one up-regulated and six down-regulated. For demethylation, 10 genes were identified (four up-regulated, six down-regulated). Acetylation involved 23 genes (seven up-regulated, 16 down-regulated), while deacetylation included six genes (two up-regulated, four down-regulated), reflecting PAC’s broad regulatory effect on gene expression during GSD (Figure 1G).

### 2.5. Search for Main Pathways Through Gene Ontology (GO) and KEGG

**Figure 3:**
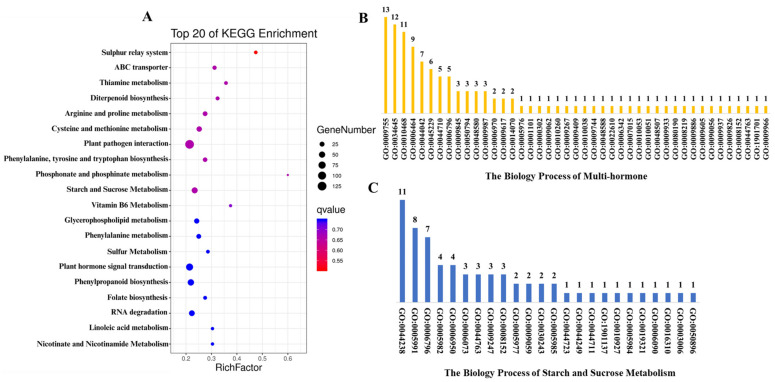
An analysis of main pathway functional enrichment: (**A**) The genes are analyzed using KEGG pathway enrichment. A bubble plot featuring gene count and Rich factor displays the most enriched pathways. (**B**) This illustration shows multiple hormones’ Gene Ontology (GO) processes, with numbers indicating gene counts involved in each biological process. (**C**) This illustration shows the Gene Ontology (GO) biological processes for starch and sucrose metabolism, with the number of genes for each process.

In the KEGG pathway analysis, our research identified two main pathways influenced by PAC in GSD: the hormone-responsive pathway and the starch and sucrose metabolism pathway. The hormone-responsive pathway, encompassing Plant Hormone Signal Transduction (ko04075) and Diterpenoid Biosynthesis (ko00904), includes 76 genes involved in the signaling of hormones such as auxin, gibberellin, cytokinin, abscisic acid, brassinosteroid, jasmonic acid, and salicylic acid, as well as in gibberellin biosynthesis critical for growth modulation. The Starch and Sucrose Metabolism pathway (ko00500) also includes 44 genes that regulate key steps in carbohydrate breakdown, impacting energy availability and structural transformations in GSD (Figure 3A). These pathways illustrate the complex gene networks driving hormone regulation and carbohydrate metabolism in response to PAC treatment, ultimately shaping seed developmental processes. Supporting these findings, Gene Ontology (GO) analysis revealed 43 distinct biological processes within the hormone-responsive pathway and 24 in the starch and sucrose metabolism pathway. Within the hormone-responsive pathway, the top three biological processes identified were the hormone-mediated signaling pathway (GO:0009755) with 13 members, the cellular macromolecule biosynthetic process (GO:0034645) with 12 members, and the regulation of gene expression (GO:0010468) with 11 members (Figure 3B). In the starch and sucrose metabolism pathway, the top three biological processes were primary metabolic process (GO:0044238) with 11 members, trehalose metabolic process (GO:0005991) with eight members, and phosphate-containing compound metabolic process (GO:0006796) with seven members (Figure 3C). These processes highlight key roles in metabolism, biosynthesis, and gene regulation, providing insights into the molecular effects of PAC on GSD.

### 2.6. Examination of Cis-Regulatory Elements Within the Promoter Regions of Multi-Hormones Genes Across Main Pathways

To better understand the transcriptional regulation and potential functions of hormone-responsive genes, we analyzed the cis-elements in the promoter regions of 76 genes, focusing on a 1.5 kb section of genomic DNA upstream of the translation start site. Using PlantCARE software (http://bioinformatics.psb.ugent.be/webtools/plantcare/html, accessed on 1 September 2024), the identified cis-elements were categorized into six groups: light-responsive, hormone-responsive, stress-related, developmental, promoter region, and site-binding elements. The most common elements were the promoter region, hormones, development, and light response cis-regulatory elements. We found 24 light-responsive cis-elements for the regulation of gene expression, including the 3-AF3 binding site, AT1-motif, ATC-motif, ATCT-motif, ACE (ACGT-containing element), AE-box, Box 4, Box II, Box III, chs-CMA1a, chs-CMA2a, chs-CMA2c, chs-Unit 1 m1, GA-motif, Gap-box, GATA-motif, G-Box, I-box, LAMP-element, L-box, MRE (Myb Recognition Element), Sp1, TCCC-motif, and TCT-motif. These elements are involved in processes like photomorphogenesis, photosynthesis, and plant development (Supplementary Figure S1A).

We also identified stress-responsive elements (LTR, MBS, W Box) and promoter-specific elements (A-box, CAAT-box, CCGTCC-box, TATA-box), with the most abundant TATA-box. This suggests that PAC may influence gene expression through stress-responsive elements and core promoter activity.

Additionally, we determined several development-related cis-elements in the promoter regions of genes related to PAC treatment. These include AACA-motif, AAGAA-motif, AC-I, AC-II, O2-site, as-1, CAT-box, CCAAT-box, circadian element, GCN4_motif, RY-element, HD-Zip 1, HD-Zip 3, MSA-like, MYB-like sequence, MYB-binding site, MYB recognition site, and MYB. These elements are essential for regulating critical processes during PAC treatment, such as growth, maturation, and the coordination of developmental pathways. Upon further analysis, these development-related elements are associated with the following transcription factor (TF) families: MYB, MADS-box, bHLH, O2, TOC1, AP2/ERF, ABI/GAMYB, bZIP, HD-Zip, and MSA-like. These TFs play significant roles in regulating critical developmental processes in plants, especially in response to PAC treatment during GSD (Supplementary Sheet S2).

Given the changes in hormone levels after PAC treatment, we further examined the hormone-responsive elements in the promoter regions. We identified 14 types of hormone-responsive cis-elements, including ABRE, ABRE3a, ABRE4, AT~ABRE, ERE, TGACG-motif, AuxRR-core, GARE-motif, P-box, TATC-box, TCA-element, TGA-box, TGA-element, and CGTCA-motif. These elements play vital roles in mediating responses to various hormones, such as abscisic acid (ABRE), ethylene (ERE), auxin (AuxRR-core), gibberellins (GARE-motif, P-box, TATC-box), and salicylic acid (TCA-element). Of particular note, 21 genes contain the P-box, six genes have the GARE motif, and 13 genes are the TATC-box—all of which are gibberellin-responsive elements influenced by PAC. This points to a complex hormonal regulatory network impacted by PAC treatment, which alters gibberellin signaling and affects the expression of genes involved in key developmental processes. Upon analyzing the hormone-responsive cis-elements identified, the following transcription factor (TF) families are associated: bZIP, MYC (bHLH), ERF/AP2, ARF, DELLA, and TGA. These TFs play essential roles in mediating hormone responses and regulating the expression of genes involved in developmental processes affected by PAC treatment (Supplementary Figure S1A).

### 2.7. Investigating Promoter Regions of Key DEGs of Starch and Sucrose Metabolism, Cell Walls, and Epigenetic Regulation

**Figure 4:**
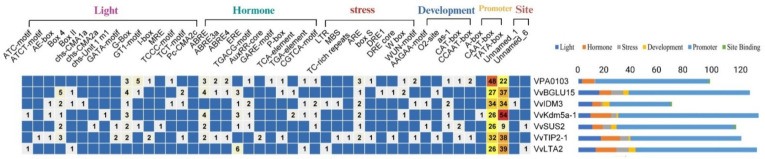
(**left**) The number of distinct cis-elements in the promoter regions of key DEGs related to starch and sucrose metabolism, cell wall remodeling, and epigenetic mechanisms. (**right**) Distribution of cis-elements across six functional categories in the promoter regions of these key DEGs.

We examined the promoter regions of key DEGs genes associated with starch and sucrose metabolism, cell wall remodeling, and epigenetic mechanisms to find cis-regulatory responsive elements. Seven genes from these pathways, showing the highest expression levels in our dataset, were identified. The cis-elements found in these promoter regions were categorized into six groups: light-responsive, hormone-responsive, stress-related, developmental, promoter region-specific, and site-binding elements. The most common elements included 16 light-responsive elements, 10 hormone-responsive elements, nine stress-responsive elements, and five development-related elements (Figure 4).

We focused primarily on the important cis-elements relevant to GSD, including ABRE, ABRE3a, ABRE4, ERE, TGACG-motif, AuxRR-core, GARE-motif, P-box, TCA-element, TGA-element, and CGTCA-motif. Upon analyzing the identified hormone-responsive cis-elements, we found associations with several TF families: bZIP, bHLH, ERF/AP2, ARF, and DELLA. These TFs are essential in mediating hormone responses and regulating gene expression in developmental processes affected by PAC treatment.

Additionally, we discovered development-related cis-elements in the promoter regions of genes associated with PAC treatment. These elements include as-1, CCAAT-box, CAT-box, O2-site, AAGAA-motif, and related transcription factors families such as AP2/ERF, MYB, MADS-box, O2, and bHLH, which influence plant growth, maturation, and various developmental pathways in response to PAC (Supplementary Sheet S2 and Figure 4).

### 2.8. Multi-Hormonal Regulation of Key Pathways in PAC-Mediated GSD

#### 2.8.1. Gibberellin (GA) Metabolism

**Figure 5:**
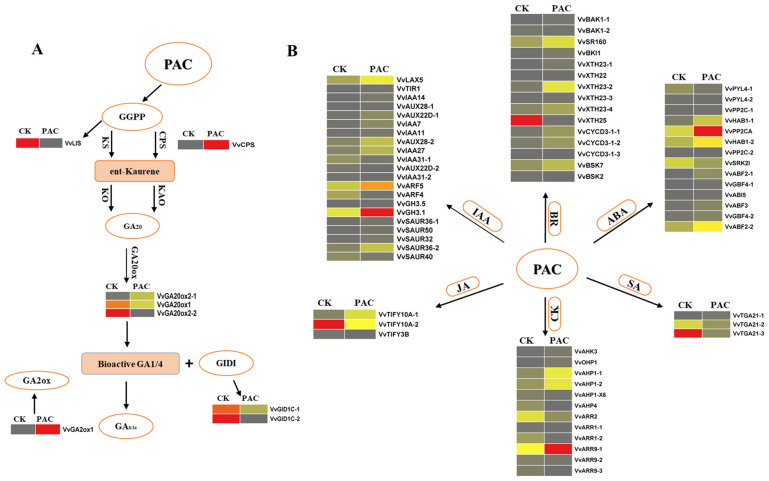
(**A**) Expression of GA pathway genes during GSD was measured using FPKM (fragments per kilobase of transcript per million mapped reads). (**B**) The interaction of PAC with other hormones, including auxin, brassinosteroids (BR), abscisic acid (ABA), cytokinins (CK), salicylic acid (SA), and jasmonic acid (JA), was assessed using FPKM (fragments per kilobase of transcript per million mapped reads).

In this exploration, five genes stand out as central to the GA biosynthesis pathway, comprising three *GA20ox* genes, one *CPS* (Copalyl Diphosphate Synthase) gene, and one P(E)-nerolidol/(E, E)-geranyl linalool synthase (*LIS*) gene. These genes play a crucial regulatory role in modulating the levels of bioactive gibberellins. Among the *VvGA20ox* genes, *VvGA20ox2-2* exhibited the highest expression levels during the CK stage, while the other two *VvGA20ox* members showed minimal expression under PAC treatment compared with CK. In contrast, *VvCPS* genes peaked in expression at the PAC stage, whereas *VvLIS* demonstrated its highest expression during the CK stage. The enzyme *VvGA2ox* is crucial in deactivating gibberellic acid (GA), playing a vital role in this inactivation process. Our investigation identified a single *VvGA2ox* gene that plays a significant role in GSD under PAC treatment. This gene, *VvGA2ox1*, demonstrated the highest expression levels during the PAC stage, underscoring its crucial function in regulating GA deactivation during this phase (Figure 5A).

#### 2.8.2. GA Signal Transduction Pathway

One GA receptor, *VvGID1C-2* (GID1A), was identified and exhibited the highest expression levels at the CK stage, indicating its crucial role in gibberellin signaling during this phase. In contrast, another GID1 family member, *VvGID1C-1*, showed intermediate expression at CK but declined under PAC treatment during GSD.

#### 2.8.3. Auxin Pathway

Our dataset revealed 21 genes associated with auxin biosynthesis, with *VvGH3.1*, a key enzyme, exhibiting the highest expression in the PAC stage and a dramatic decline in the CK stage. *VvARF5*, a transcriptional regulator known to repress a series of auxin responses, emerged as the most highly expressed gene among *VvARF4* family members, while *VvARF4* itself displayed lower expression at the CK stage. Furthermore, we identified the auxin transporter *VvLAX5* and the auxin receptor *VvTIR1*. *VvLAX5* maintained moderate expression throughout both PAC and CK stages, contrasting with *VvTIR1*, which showed no detectable expression at either stage. Several exhibited minimal expressions among the 10 AUX/IAA proteins identified during both stages. Five *VvSAUR* genes were identified, solidifying their importance in the auxin pathway alongside *VvARFs*, AUX/IAA proteins, and *VvSAUR40* and *VvSAUR36*, which exhibited high transcription throughout GSD.

#### 2.8.4. BR Pathway

For brassinosteroid biosynthesis, 15 genes were identified, in which medium expression was shown by the systemin receptor VvSR160 at the PAC stage, while it showed a decline at the CK stage. In comparison, two associated receptor genes showed initial expression at both stages, including *VvBAK1-1* and *VvBAK1-2*. Of the two proteins of the *VvBSK* family, only one, i.e., *VvBSK-1*, showed minimal dominancy in the PAC and a decline in the CK stage. The other important gene, *VvBKI1*, showed some expression in the PAC stage. Six members of the *VvTCH4* gene were found, of which *VvXTH25* showed high expression at the CK stage, while other members were initially expressed at both stages. Three members of gene *VvCYCD3* shift their significant expression from PAC to CK, including *VvCYCD3-1-1*, *VvCYCD3-1-2*, and *VvCYCD3-1-3.*

#### 2.8.5. ABA Pathway

We found 14 genes for ABA biosynthesis, of which five were members of the *VvPP2C* gene. During GSD, *VvPP2CA* was significantly expressed at the PAC stage and less so at the CK stage, whereas other members of *VvPP2C* were represented at both stages. Additionally, one gene, *VvSRK2I*, was discovered to exhibit both a significant fall and middle expression throughout the CK stage. Two ABA receptors were identified; *VvPYL4-1* had the lowest expression at the CK stage and dropped significantly to the PAC stage, while *VvPYL4-2* displayed only a trim expression level in the CK stage. Six *VvABF* genes were also found and initially expressed in both phases.

#### 2.8.6. CK Pathway

For CK biosynthesis, we discovered 12 genes, with one CK receptor, *VvAHK3,* being identified. The receptor exhibited the lowest expression during the PAC stage. Among the five *VvAHP* member genes identified, *VvAHP1-1* and *VvAHP1-2* displayed moderate expression during the PAC stage and the lowest during the CK stage. The remaining members of *VvAHP* initially expressed themselves at both stages. Additionally, we detected three Type-A response regulators, *VvAAR-A*, and three Type-B response regulators, *VvAAR-B. VvARR9-1* demonstrated the highest expression during the PAC stage and moderate expression during the CK stage, while other members of *VvAAR-A* exhibited the lowest expression during the CK stage. *VvARR2* showed moderate expression during the CK stage and a significant decline during the PAC stage. The other two members of *VvAAR-B* displayed initial expression during the CK stage.

#### 2.8.7. JA Pathway

We identified three genes in the JA pathway (TIFY family), including *VvTIFY10A-1*, *VvTIFY10A-2,* and *VvTIFY3B*. *VvTIFY10A-2* showed high expression during the CK stage, followed by a significant decline in the PP stage. Meanwhile, *VvTIFY10A-1* and *VvTIFY3B* maintained moderate expression levels across both stages. These expression trends suggest that *VvTIFY10A-2* may respond more to PAC treatment, while *VvTIFY10A-1* and *VvTIFY3B* are likely involved in stable, ongoing developmental processes.

#### 2.8.8. SA Pathway

We identified three members of the *TGA2.2* family of genes in the salicylic acid (SA) pathway: *VvTGA21-1, VvTGA21-2*, and *VvTGA21-3. VvTGA21-3* exhibited the highest expression levels during the CK stage, indicating its potential role in the grapevine’s baseline defense mechanisms. However, we observed a significant decline in its expression at the PAC stage, suggesting that the hormonal modulation may affect its function in stress response. In contrast, *VvTGA21-1* and *VvTGA21-2* maintained moderate expression levels at both stages, which may imply a more stable regulatory role in the SA pathway (Figure 5B).

### 2.9. Starch and Sucrose Metabolism

**Figure 6:**
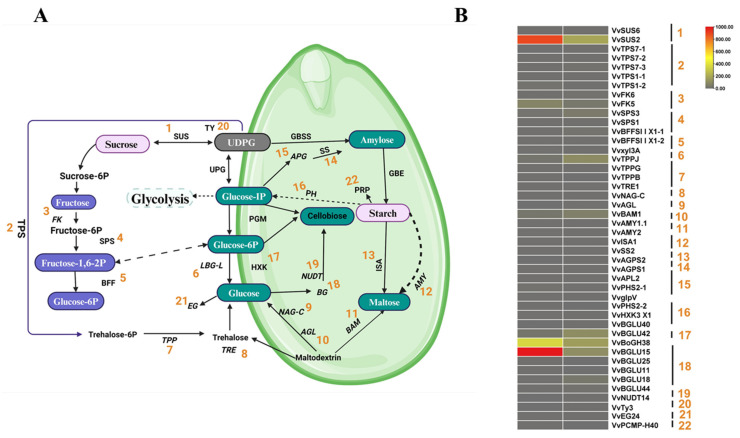
Starch and Sucrose Metabolism Pathway. (**A**) Model and key components of starch and sucrose metabolism in GSD (**B**) Expression of genes in the Starch and sucrose metabolism pathway during GSD, with gene expression levels quantified in FPKM (Fragments Per Kilobase of transcript per Million mapped reads.

The present study identified 44 genes associated with starch and sucrose metabolism during GSD under PAC treatment. *VvSUS2* (sucrose synthase 2) and *VvBGLU15* (beta-glucosidase 15) exhibited high expression levels during the CK stage but dramatically declined in the PAC stage. Similarly, *VvBoGH38* (beta-glucosidase 38) demonstrated moderate expression in the CK stage, which decreased to minimal levels in the PAC stage. In contrast, *VvFK5* (fructokinase 5) maintained consistently low expression across both stages. Additionally, genes such as *VvTPPJ* (trehalose-6-phosphate phosphatase J), *VvBAM1* (beta-amylase 1), and *VvBGLU42* (beta-glucosidase 42) displayed low expression specifically during the PAC stage. Among the 44 genes, 24 were up-regulated, while 20 were down-regulated, highlighting the significant impact of PAC treatment on starch and sucrose metabolism in GSD (Figure 6A,B).

### 2.10. Unveiling the Dynamics of DNA Methylation and Demethylation at Their Expression Levels

**Figure 7:**
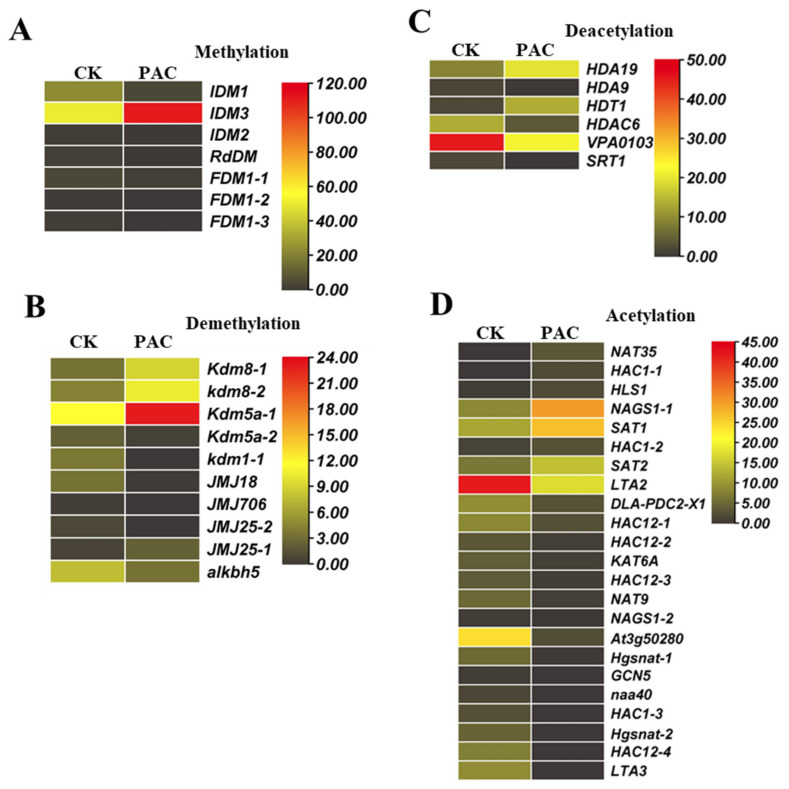
Expression profiles of epigenetic modifications during GSD under PAC treatment. (**A**) The expression changes of methylation relation genes indicate methylation might be involved in the modulation of GSD under PAC treatment. (**B**) Demethylation highlights genes responsible for removing methyl groups. (**C**) Deacetylation depicts the gene expression levels involved in histone deacetylation. (**D**) Acetylation: representing the expression of genes regulating histone acetylation.

In grapes, a methyl group is added to DNA in DNA methylation, which acts on the most crucial stage, i.e., CpG dinucleotide sites in *Vitis vinifera*. It affects several biological processes by controlling gene expression, silencing transposons, and reacting to stress. On the other hand, demethylation is removing methyl groups from DNA to reverse the methylation process. It behaves differently, passively or by enzymatic mechanisms, during DNA replication. According to the analysis, it was revealed that a total of 17 genes fall into the category of DNA methylation and demethylation, of which seven genes belong to the former category. In comparison, 10 genes belong to the latter category. The most important and only gene found to be involved in methylation was *IDM3*, which resulted in the highest expression during the PAC stage as opposed to the CK stage. The other six genes of the category DNA methylation showed the slightest expression in both the CK and PAC stages. The expression patterns of the demethylation genes were also significant to consider for a better understanding. An essential gene of the class demethylation genes, i.e., *KDM5A-1*, showed the highest expression during the PAC stage compared with CK. In PAC, moderate expression was observed from two genes, *KDM8-1* and *KDM8-2*, while this expression decreased in CK. The only gene, *JMJ706*, that belongs to the demethylation of DNA showed the least amount of expression in both CK and PAC treatments compared with the other six genes (Figure 7A,B).

### 2.11. Expression Profile and Pattern Analysis of Acetylation and Deacetylation Enzymes

Gene expression changes and epigenetic modifications are dynamic in regulating several developmental processes in grape seeds, including cell division, proliferation, and maturation. The regulation of chromatin structure and gene expression patterns during seed development is aided by the acetylation and deacetylation of histones by enzymes such as histone acetyltransferases (HATs) and histone deacetylases (HDACs). The investigation showed that 29 genes: 23 acetyltransferase proteins and 06 deacetylase proteins, fit into the categories of acetylation and deacetylation. Out of the 23 acetyltransferase proteins analyzed, the most significant gene identified was LTA2, which exhibited the highest expression level during the CK stage compared with the PAC stage. *NAGS1-1, SAT1*, and SAT2 displayed intermediate expression levels during the PAC stage, whereas during the CK stage, *At3g50280* had intermediate expression. Furthermore, one acetyltransferase protein gene exhibited no expression levels in either stage. The remaining 17 acetyltransferase protein genes exhibited the lowest expression levels in both the CK and PAC stages. Regarding deacetylation, *VPA0103* expressed most at the CK stage compared with PAC. The genes encoding the remaining five deacetylase proteins had the lowest expression levels in the CK and PAC stages (Figure 7C,D).

### 2.12. Genes Encoding Differentially Expressed Cell Wall Remodeling Enzymes

**Figure 8:**
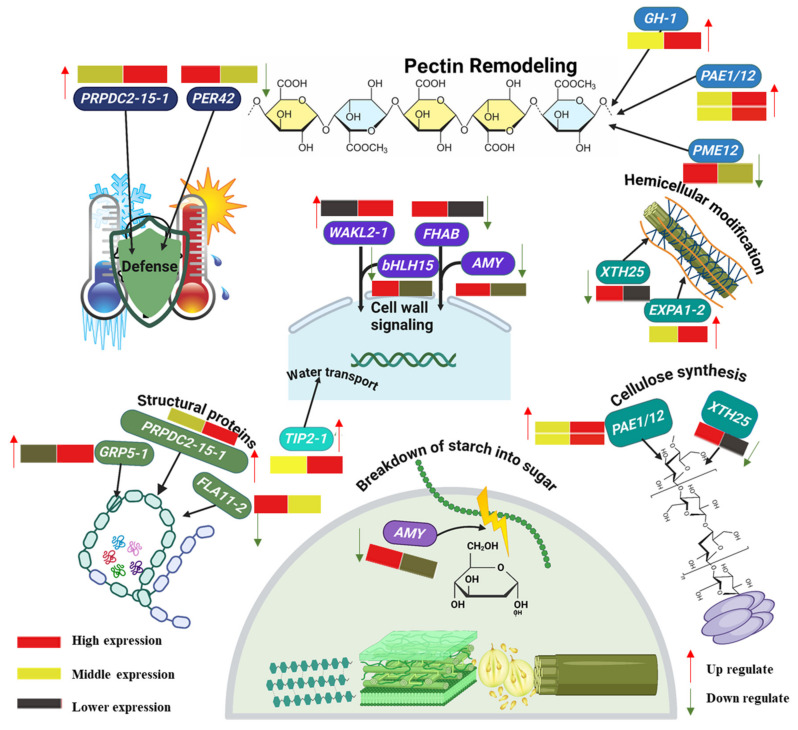
Comprehensive overview of cell wall modifying gene expressions across various families. The heat map represents the expression of specific cell wall-modified genes, with red arrows indicating up-regulation and green arrows indicating down-regulation.

In our study on GSD, we examined 101 cell wall modifying genes across 14 families under the influence of PAC. These families include XTH (Xyloglucan Endotransglucosylase/Hydrolase), involved in cell wall remodeling; bHLH (Basic Helix-Loop-Helix), a family of transcription factors regulating development and stress responses; AMY (Amylase), which breaks down Starch for energy; EXPA (Expansin), proteins facilitating cell expansion; FLA (Fasciclin-Like Arabinogalactan), involved in cell adhesion and signaling; GRP (Glycine-Rich Proteins), contributing to cell wall integrity; PRP (Proline-Rich Proteins), strengthening the cell wall and aiding in defense mechanisms; WAKL (Wall-Associated Kinase-Like), proteins involved in cell wall signaling and stress responses; TIP (Tonoplast Intrinsic Proteins), aquaporins facilitating water transport; PME (Pectin Methylesterase), enzymes modifying pectin to affect cell wall rigidity; GH (Glycoside Hydrolase), enzymes involved in cell wall degradation and remodeling; FHAB (Fasciclin Homolog A and B), involved in cell adhesion and communication; PAE (Pectin Acetylesterase), enzymes influencing cell wall structure; and PER (Peroxidase), involved in lignin biosynthesis and defense. Our findings reveal that PAC significantly modulates cell wall remodeling during GSD, leading to the up-regulation of eight key genes and the down-regulation of seven key genes, highlighting the intricate balance of cell wall dynamics influenced by this growth regulator (Figure 8).

### 2.13. Identification of Transcription Factor (TF) Families Involved in PAC-Mediated GSD

**Figure 9:**
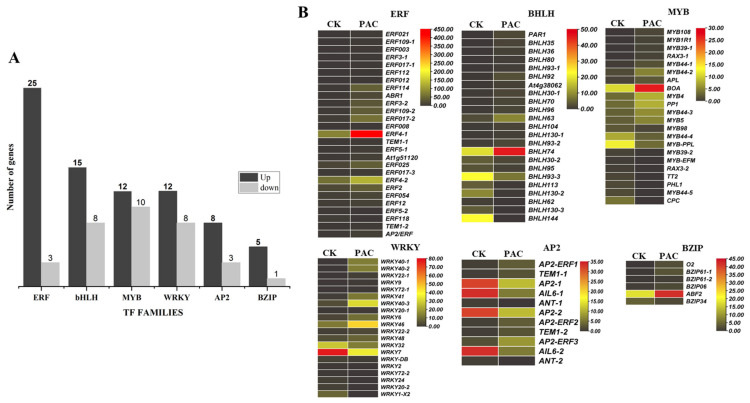
(**A**) Identification of transcription factors exhibiting up-regulation and down-regulation. (**B**) Expression patterns of transcription factor families (TFFs) involved in PAC-mediated GSD.

Analysis of transcription factor (TF) families identified 110 TFs with significant changes in expression, with 77 up-regulated and 33 down-regulated. When comparing six specific TF families, we found different patterns of regulation. The MYB family had 12 TFs up-regulated and 10 down-regulated, while the BZIP family had five up-regulated and one down-regulated. The ERF family showed strong up-regulation, with 25 TFs up-regulated and only three down-regulated. The BHLH family had 15 TFs up-regulated and eight down-regulated. The AP2 family had eight TFs up-regulated and three down-regulated. Finally, the WRKY family had 12 TFs up-regulated and eight down-regulated (Figure 9A).

Peak expression was observed in specific genes across different families. The MYB family showed the highest expression at the PAC stage with the gene BOA. Similarly, the ERF family exhibited peak expression at the PAC stage with ERF4-1. For the bHLH family, bHLH74 reached its highest expression at the PAC stage, and the bZIP family’s ABF2 also showed peak expression at the PAC stage. In contrast, the AP2 and WRKY families had peak expression at the CK stage. Specifically, the AP2 family included genes AP2-1, AP2-2, AIL6-1, and AIL6-2, while the WRKY family had WRKY7 showing peak expression uniquely at the CK stage. Thus, aside from WRKY7, which peaked at the CK stage, all other representative genes showed their highest expression at the PAC stage. Four transcription factor families showed peak expression at the PAC stage (Figure 9B).

### 2.14. Validation of Main Pathway Gene Expressions Using RT-qPCR Analysis

**Figure 10:**
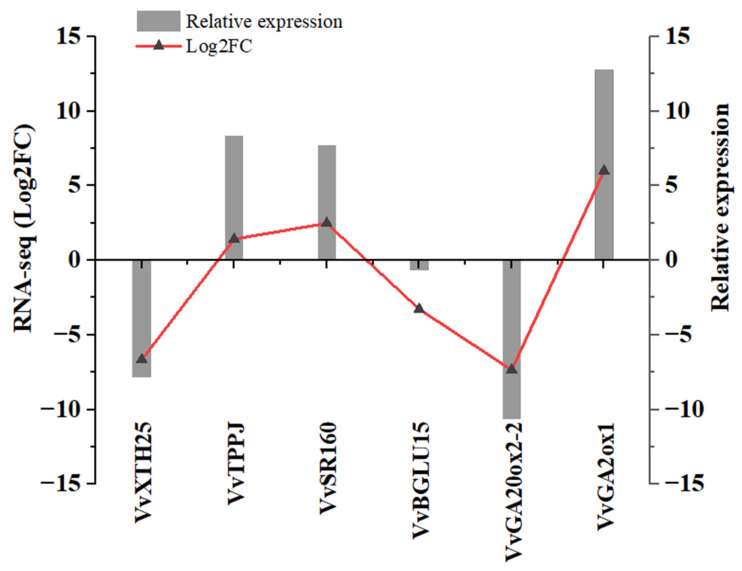
Data analysis of main pathway genes between RT-qPCR in the application of PAC in GSD.

To validate the gene expression patterns identified through RNA-Seq, six core genes *VvGA2ox1*, *VvGA20ox2-2*, *VvXTH25*, *VvSR160*, *VvTPPJ*, and *VvBGLU15* were selected for further analysis using RT-qPCR (Figure 10). These genes were chosen for their high expression levels during GSD and critical roles in the main pathways influenced by PAC treatment. The RT-qPCR results revealed expression profiles closely aligned with the RNA-Seq data, confirming that the RNA-Seq approach reliably captures the relative expression levels of these genes in GSD under PAC influence. This consistency underscores the accuracy and robustness of RNA-Seq as a method for evaluating gene expression dynamics in response to PAC.

## 3. Discussion

### 3.1. Role of PAC as a Gibberellin Inhibitor in Shaping Hormonal Interplay in GSD

Seed development is a highly coordinated and dynamic process regulated by various hormonal, genetic, and metabolic pathways [15]. In the presence of PAC, a plant growth regulator inhibiting gibberellin biosynthesis, these pathways are altered, particularly during critical stages such as stone hardening. The stone-hardening stage is a complex phase in seed development, where both cell wall modifications and hormonal changes are carefully coordinated [16]. While PAC inhibits GA, slowing growth, it simultaneously increases the activity of other hormones, such as auxin, which helps regulate the cellular processes necessary for tissue differentiation. The elevated auxin levels seen during PAC treatment may stimulate the production of specific cell wall components, which contribute to the firmness and mechanical strength needed for seed maturation [17].

PAC is a synthetic plant growth regulator widely recognized for inhibiting gibberellin biosynthesis, an essential hormone pathway that, in coordination with auxin distribution, can influence cell elongation and division depending on cell fate and auxin transport modulation [18]. PAC profoundly impacts plants’ physiological and developmental processes by reducing gibberellin levels. This regulation results in controlled growth and a more compact plant structure and influences other essential developmental stages, such as seed and fruit maturation [19]. GA biosynthesis has two main stages: the early stage with CPS, KS, KO, and KAO, and the late stage with *GA20ox* and *GA3ox* [20]. Gibberellins are plant hormones that enhance growth and development [21]. As GA levels rise, the expression of late GA biosynthesis genes is reduced. This regulation is crucial because GA can result in unchecked growth and developmental issues [22]. By limiting GA synthesis at elevated concentrations, plants can maintain homeostasis and avoid the excessive buildup of these hormones. Nonetheless, a negative feedback mechanism driven by GA has been suggested. This mechanism involves down-regulating late GA biosynthesis genes while up-regulating the GA-deactivating gene GA2ox. This helps to ensure that GA levels remain balanced [23]. We identified five key genes involved in the GA biosynthesis pathway: three *GA20ox* genes, one *CPS* (Copalyl Diphosphate Synthase) gene, and one P(E)-nerolidol/(E, E)-geranyl linalool synthase (*LIS*) gene. These genes may play a significant regulatory role in adjusting the levels of bioactive gibberellins [24]. Additionally, we found that the enzyme *VvGA2ox* is crucial for deactivating gibberellic acid (GA), playing a vital role in this inactivation process. Our investigation identified a single *VvGA2ox* gene significantly contributing to GSD under PAC treatment. This gene, *VvGA2ox1*, demonstrated the highest expression levels during the PAC stage, underscoring its crucial function in regulating GA deactivation during this phase. It might indicate a compensating mechanism in response to PAC. In addition to GA biosynthesis, long- and short-distance GA movements are also critical for developmental processes. One GA receptor, *VvGID1C-2* (GID1A), was identified and exhibited the highest expression levels at the CK stage, indicating its crucial role in gibberellin signaling during this phase. In contrast, another member of the *GID1* family, *VvGID1C-1*, showed intermediate expression at the CK stage but declined under PAC treatment during GSD. Our results suggest that low GA production from PAC treatment activates a complex GA biosynthesis and signaling system, helping to mitigate the effects of reduced GA levels and support developmental processes. PAC treatment in GSD induces cross-talk among auxin, ABA, BR, CK, SA, and JA, harmonizing growth and stress responses to support development despite inhibited GA synthesis [14,25,26,27,28]. In contrast, IAA is vital for regulating various aspects of growth and development, including cell elongation, root formation, and fruit development [29]. The elevation of IAA levels in the presence of PAC suggests a compensatory mechanism where auxin pathways might be up-regulated in response to suppressed gibberellin activity. By converting free IAA into these conjugated forms, *GH3.1* serves as a mechanism to modulate auxin levels, especially in response to environmental cues or developmental stages [30]. The expression of *GH3.1* varies across different tissues and developmental stages, indicating its role in tissue-specific and developmental-regulated processes [31]. It has been observed that the expression levels of *VvGH3.1* under osmotic stress can significantly change, indicating its adaptive responses. As a key enzyme in auxin conjugation, *VvGH3.1* exhibited the highest expression during the PAC stage and a marked decrease during the CK stage. This suggests that auxin activity might initially increase due to PAC application but later diminish as other regulatory mechanisms take over. *GH3.1* has been implicated in various growth processes within grapevine, such as fruit development and ripening [32,33]. The regulation of auxin levels through *GH3.1* may influence processes like apical dominance, root development, and overall plant architecture.

Brassinosteroids (BRs) are crucial phytohormones that promote cell elongation, division, and overall plant growth, primarily by modulating auxin effects [34]. They enhance tolerance to abiotic stresses, significantly adapting plants to fluctuating environmental conditions [35]. BRs were shown to act synergistically with auxin to regulate cell wall extensibility and growth processes [36]. Our results align with previous findings that demonstrate the role of BR in enhancing cell wall extensibility, primarily through the regulation of xyloglucan endotransglucosylase genes. Specifically, the expression of xyloglucan endotransglucosylase/hydrolase protein 23 (*XTH23*) was observed to be modulated by BR, indicating a potential mechanism through which BR regulates growth and structural development in grapevine seedlings [37,38].

Furthermore, the interaction between BR and PAC presents an interesting dynamic. PAC acts as a gibberellin inhibitor, leading to altered BR signaling pathways. This interaction may explain the observed reduction in *VvXTH25* gene expression under PAC treatment. These findings suggest that BR promotes growth but the efficacy may be diminished in environments where GA synthesis is inhibited, highlighting a critical balance between these hormonal pathways in plant development [39].

Protein phosphatases 2C (*PP2Cs*) are crucial negative regulators in the ABA signaling pathway, influencing how plants respond to environmental stresses [40]. Our study investigated protein phosphatase 2C 37 (*PP2C37*) and its expression during GSD under PAC treatment. PAC, a plant growth regulator, suppresses gibberellin (GA) synthesis, enhancing ABA levels and shifting the plant’s hormonal balance. *PP2C37’s* role in stress response is well-documented in rice, where its homolog OsPP2C09 acts as a negative regulator in the ABA pathway, helping to moderate ABA responses and enhance drought tolerance [41]. In our findings, the grape homolog, *VvPP2CA*, showed significantly increased expression in grape seeds under PAC treatment. This suggests that VvPP2CA may be instrumental in helping grape seeds adjust to PAC-induced hormonal changes, likely boosting their sensitivity to ABA and improving their resilience to stress conditions.

The *ORR9* gene, also known as *VvARR9-1* in grapes, plays a critical role in the cytokinin signaling pathway, which is essential for plant growth and development [42]. This gene encodes a type-A response regulator protein, which acts in a two-component signaling system commonly associated with cytokinin responses. *ORR9* expression is notably higher during the PAC treatment stage in GSD, indicating its significant role in promoting seed maturation under these conditions. Predominantly active in the chalaza of developing seeds, *ORR9* works closely with phosphotransfer proteins like *AHP2*, *AHP3*, and *AHP5*, which mediate the transfer of phosphate groups essential for cytokinin signaling. This interaction enables *ORR9* to regulate the expression of cytokinin-responsive genes, orchestrating cell division and developmental processes in seeds [43]. By modulating cytokinin responses, *ORR9* facilitates favorable conditions for the growth and maturation of grape seeds, thereby highlighting its regulatory role in seed development [44].

The salicylic acid pathway (SA) is crucial in plant stress responses and intersects with various hormone signaling pathways, including *TGA2.2* [45]. Potentially influenced by salicylic acid levels, the regulation of TGA2.2 may affect grape development, particularly in defense against pathogens and in stress adaptation processes during seed development [46]. In a previous study, tobacco bZIP, transcription factor *TGA2.2*, is implicated in plant defense mechanisms and development [47]. It regulates responses to various stresses, including developmental processes in grapes, particularly under conditions where growth regulators like PAC are used. Recently, it was found that the decreased expression of *VvTGA2.2* in PAC suggests that while PAC enhances certain aspects of seed quality, it may simultaneously limit the activation of specific defense mechanisms regulated by *TGA2.2*, thereby influencing the resilience and performance of the developing seed [48].

*VvTIFY10A-2* plays a critical role in GSD by modulating the jasmonic acid (JA) pathway, which influences seed maturation, seedling vigor, and responses to environmental stresses [49,50]. TIFY proteins, including *TIFY10A*, regulate key genes in the JA signaling pathway, enhancing the plant’s resilience to various stresses, such as drought and salt, which are particularly important during seed development [50]. Under PAC treatment, the down-regulation of *VvTIFY10A-2* can negatively impact the JA pathway. Lower levels of *VvTIFY10A-2* reduce JA sensitivity, potentially compromising the plant’s ability to manage developmental processes like seed maturation and germination efficiently. Additionally, down-regulated *VvTIFY10A-2* impairs other stress response pathways, underscoring its essential role in supporting overall plant health and resilience during crucial stages of development [51].

### 3.2. PAC-Induced Changes in Starch and Sucrose Metabolism: Implications for GSD

The starch and sucrose regulatory pathways are essential for maintaining sugar balance and energy homeostasis in plant growth [52]. Sucrose is a substrate for storage reserves and a primary energy source for seed development [53,54] The present study examined the down-regulation of key enzymes such as sucrose synthase (*VvSUS2*) and beta-glucosidase 15 (*VvBGLU15*) observed in the presence of PAC, which suggests significant alterations in these metabolic pathways [55], and these variations can result into the reduction of energy sources, potentially affecting seed development. Sucrose synthase, crucial for converting sucrose to UDP-glucose and fructose, may decrease essential metabolite availability, impacting the energy production required for seed growth [56]. Similarly, the down-regulation of *VvBGLU15*, responsible for hydrolyzing glucosidic bonds to release glucose, could further disrupt sugar metabolism and energy homeostasis in grape seeds [57]. The reduced activity of these enzymes might lead to an accumulation of non-metabolized carbohydrates, shifting the metabolic flux and potentially conserving energy during stress conditions induced by PAC [58]. These adjustments highlight the intricate balance between hormone signaling, carbohydrate metabolism, and seed development, where PAC plays a pivotal role in modulating these processes [59].

### 3.3. Regulation of Cell Wall Enzymes Genes by PAC

In *Arabidopsis* and tomato seed germination, PAC induces the expression of several genes encoding enzymes involved in cell elongation and cell wall remodeling, such as XTH, PME, expansins, pectin lyases, and aquaporins [60,61]. Our study revealed significant differential expression in many cell wall remodeling genes. We identified highly expressed bHLH, PER, and TIP family genes that play pivotal roles in PAC regulation in GSD. Interestingly, these gene families were previously studied in soybean embryonic axes during germination under the influence of PAC [62]. Furthermore, our investigation highlighted differential expression in 19 peroxidase genes alongside genes associated with pectin metabolism influenced by PAC. Additionally, we identified other crucial cell wall-related genes essential for cell proliferation, including arabinogalactan proteins (AGPs), fasciclin-like AGPs, hydroxyproline (Hyp)-rich glycoproteins, and proline or glycine-rich proteins [63,64,65]. Significantly, the 53 out of 101 cell wall genes up-regulated by PAC underscore the well-established role of gibberellic acid (GA) in promoting cell wall loosening and remodeling to facilitate embryo expansion during GSD [62].

### 3.4. Regulatory Roles of Transcription Factors in PAC-Affected GSD

**Figure 11:**
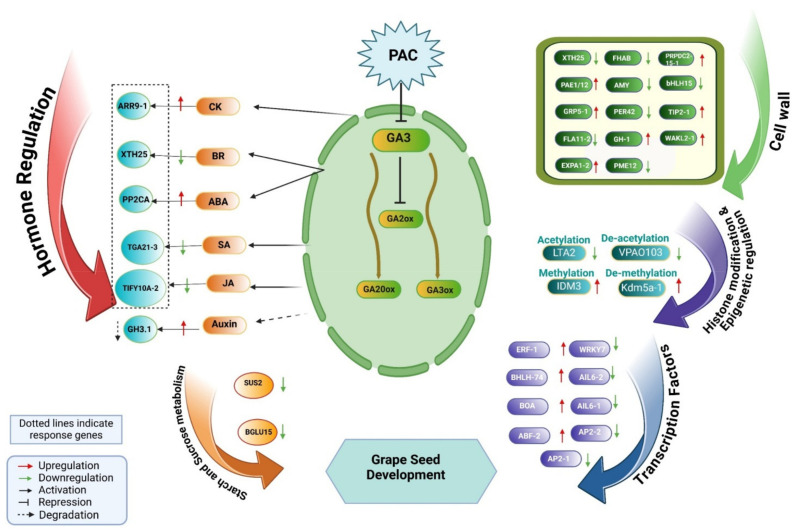
Integrative Regulatory Network: PAC Impact on GSD.

The differential expression of 110 transcription factors (TFs) in response to PAC treatment highlights the complex regulatory network involved in GSD. The notable up-regulation of ERF, MYB, BZIP, and bHLH family members suggests their crucial roles in mediating PAC’s effects on seed development [62]. The contrasting down-regulation of AP2 family members may indicate a potential mechanism for altering seed size or mass. At the PAC treatment, the peak expression of specific genes like BOA, ERF4-1, ABF2, and bHLH74 points to their potential as key mediators of PAC’s effects. Interestingly, the unique expression pattern of *VvWRKY7* peaking at the CK stage suggests its involvement in early developmental processes [66,67]. The minimal expression of most TFs at both stages underscores the specificity of the transcriptional response to PAC treatment. These findings collectively suggest that PAC activates targeted transcriptional programs, primarily mediated by ERF, MYB, BZIP, and bHLH families; this transcriptional reprogramming likely underlies the observed effects of PAC in GSD, offering potential targets for future breeding programs aimed at manipulating seed traits in grapes [62] (Figure 11).

## 4. Materials and Methods

### 4.1. Plant Materials

*Vitis vinifera* cv. ‘Wink’ grapevines were cultivated at the Lishui Experimental Farm of Nanjing Agricultural University, Jiangsu Province, China, under standard field management practices. The region has a subtropical monsoon climate, with an average annual temperature of 16.4 °C and 1980 h of sunshine. Five-year-old grapevines, trained on a vertical shoot-positioning system, were spaced at 1.5 m × 2.5 m. PAC solution (50 mg·L^−1^, Sigma-Aldrich, ≥98% purity) with 0.1% Tween-80 was freshly prepared and applied by immersing grape clusters for 30 s at 10 days after flowering. Control clusters were treated with water containing 0.1% Tween-80. Seed samples were collected 30–50 days after flowering from 10 clusters per replicate, pooled into three biological replicates, frozen in liquid nitrogen, and stored at −80 °C

### 4.2. Analysis of Differentially Expressed Genes

Read count data were used as input data to analyze differentially expressed genes in the software package DESeq2 3.20, accessed on 20 August 2024. The analysis was divided into three parts: (1) normalizing read counts, (2) calculating hypothesis testing probabilities (*p*-values) based on the model, and (3) performing multiple hypothesis tests and corrections to obtain the false discovery rate (FDR). Based on this analysis, FDR < 0.01 and |log2FC| ≥ 1 (where FC is the fold change) were screened as significantly different and categorized as differentially expressed genes (DEG). Transcripts |log2FC| < 0.25 were considered to have no change in expression level. Other transcripts (0.25|log2FC| < 1) were considered “slightly up-regulated” or “slightly down-regulated”.

### 4.3. Chromosomal Localization, Phylogenetic Analysis, and Gene Structure Analysis

The information on the location of each hormone-responsive gene was obtained from the whole genomic sequence according to the Grape genome database (http://genomes.cribi.unipd.it/grape/, accessed on 23 August 2024). Then, the map was drafted using MapGene2 Chromosome (http://mg2c.iask.in/mg2c_v2.0/, accessed on 25 August 2024). Phylogenetic analysis was performed using MEGA software11.0, accessed on 26 August 2024), where gene sequences were aligned, and a phylogenetic tree was constructed using the Neighbor-Joining method. The resulting tree was exported in Newick format. Gene structure analysis was conducted on GSDS 2.0 (https://gsds.gao-lab.org/, accessed on 28 August 2024) by uploading the Newick file alongside genomic sequences, allowing for visualization of the exon-intron organization aligned with the phylogenetic tree.

### 4.4. Conserved Domain Analysis and Cis-Acting Elements Analysis

Conserved domains were identified using the Conserved Domain Database (CDD) from NCBI (https://www.ncbi.nlm.nih.gov/Structure/cdd/wrpsb.cgi, accessed on 29 August 2024). The downloaded CDD file was analyzed in TBtools using the “Conserved Domain Analysis” tool to annotate functional motifs in the target gene sequences. The 1500 bp region upstream of hormone-responsive and other key genes was defined as putative promoter sequences. PlantCARE tool (http://bioinformatics.psb.ugent.be/webtools/plantcare/html, accessed on 1 September 2024) was used to predict the cis-acting elements.

### 4.5. Functional Annotation and Differentially Expressed Genes

GO and KEGG pathway analyses were conducted using the Gene Ontology database (http://geneontology.org, accessed on 2 September 2024) and the Kyoto Encyclopedia of Genes and Genomes (KEGG) database to categorize and interpret DEGs. Heatmap analysis was performed in TBtools v2.056, visualizing normalized expression data and clustering genes based on expression patterns across different conditions.

### 4.6. RNA Extraction and Quantitative PCR Analysis

Using a modified CTAB method, RNA was extracted from grape samples [68]. Hifair^®^ II 1st Strand cDNA Synthesis SuperMix (Yeasen, Shanghai, China) was then used to reverse transcribe this RNA to cDNA. The qRT-PCR process utilized Hieff^®^ qPCR SYBR Green Master Mix (Yeasen, Shanghai, China). Primers were designed in Primer 3.0 and verified using NCBI’s online tools (https://www.ncbi.nlm.nih.gov/, accessed on 4 September 2024). Each 20 µL qRT-PCR reaction contained 10 µL of 2× SYBR Green mix, 2 µL of cDNA, and 0.2 µM of each primer. Actin was used as a reference gene to normalize the data. Each test was performed in triplicate, and relative expression was calculated by the 2^−ΔΔCT^ method [69]. All primers are provided in the Supplementary Sheet S3.

## 5. Conclusions

Our study highlights the significant impact of PAC on GSD by modulating key molecular pathways. PAC treatment led to the extensive reprogramming of gene expression, including the up-regulation of auxin, ABA, and cytokinin-related genes and the down-regulation of gibberellin biosynthesis genes, confirming its role as a GA inhibitor. Notably, PAC altered the expression of genes involved in starch and sucrose metabolism, cell wall remodeling, and transcription factor regulation, reflecting a complex regulatory network. The analysis of cis-regulatory elements further revealed critical motifs associated with development, stress, and hormonal signaling, underscoring their role in GSD. These findings provide valuable insights into PAC’s multifaceted effects, offering potential strategies for improving grapevine development through the targeted manipulation of hormonal and molecular pathways.

## Data Availability

This published article and its Supplementary Materials include all data generated or analyzed during this study.

## Supplementary Materials

The following supporting information can be downloaded at: https://www.mdpi.com/article/10.3390/ijms26031102/s1.
