# Supplementary figures and images for: Characterization of Main Responsive Genes Reveals Their Regulatory Network Attended by Multi-Biological Metabolic Pathways in Paclobutrazol (PAC)-Modulated Grape Seed Development (GSD) at the Stone-Hardening Stage
Source: Int J Mol Sci. 2025 Jan 27;26(3):1102. doi: 10.3390/ijms26031102 (PMC11817196; doi:10.3390/ijms26031102)

A

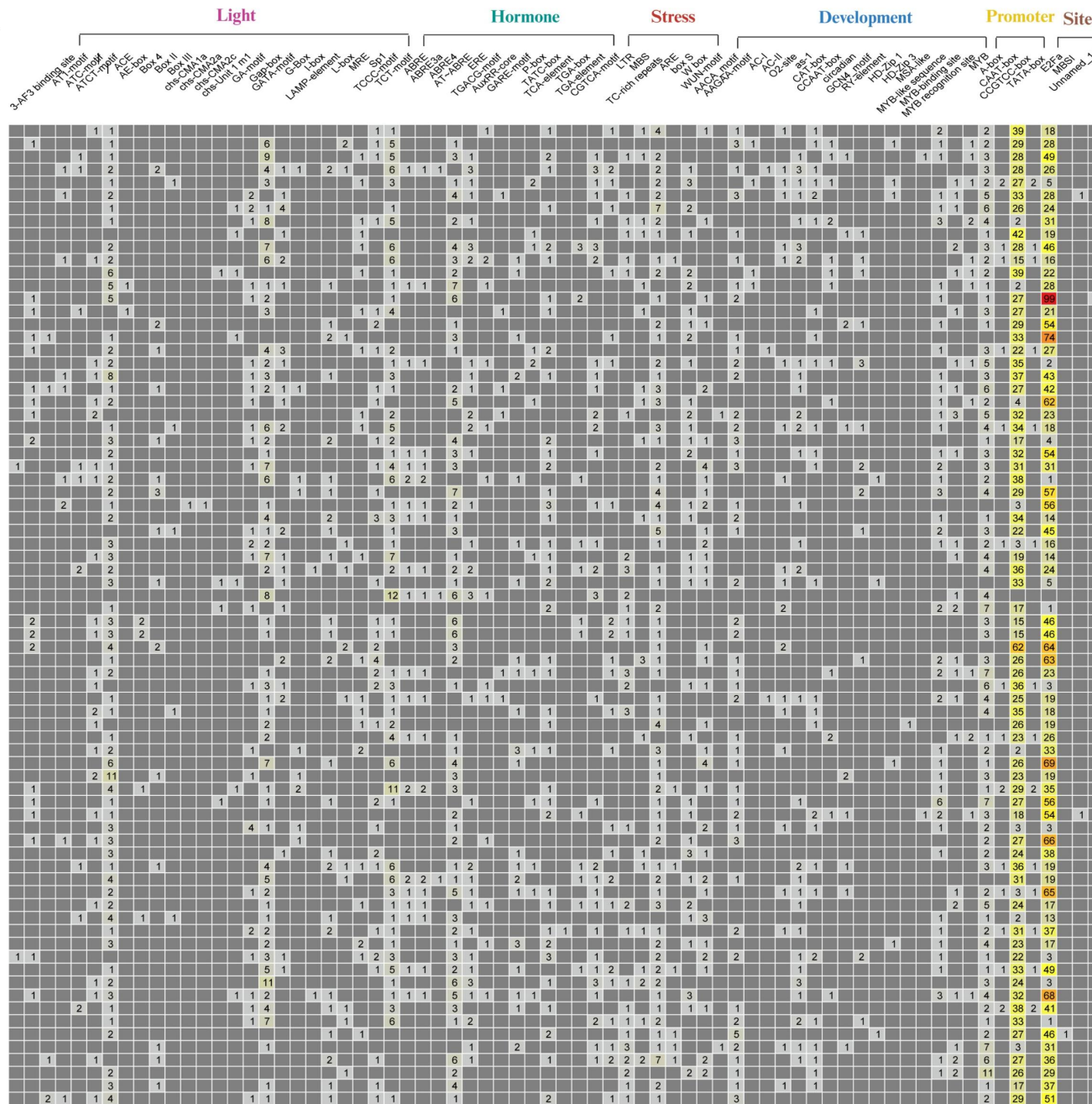

# B

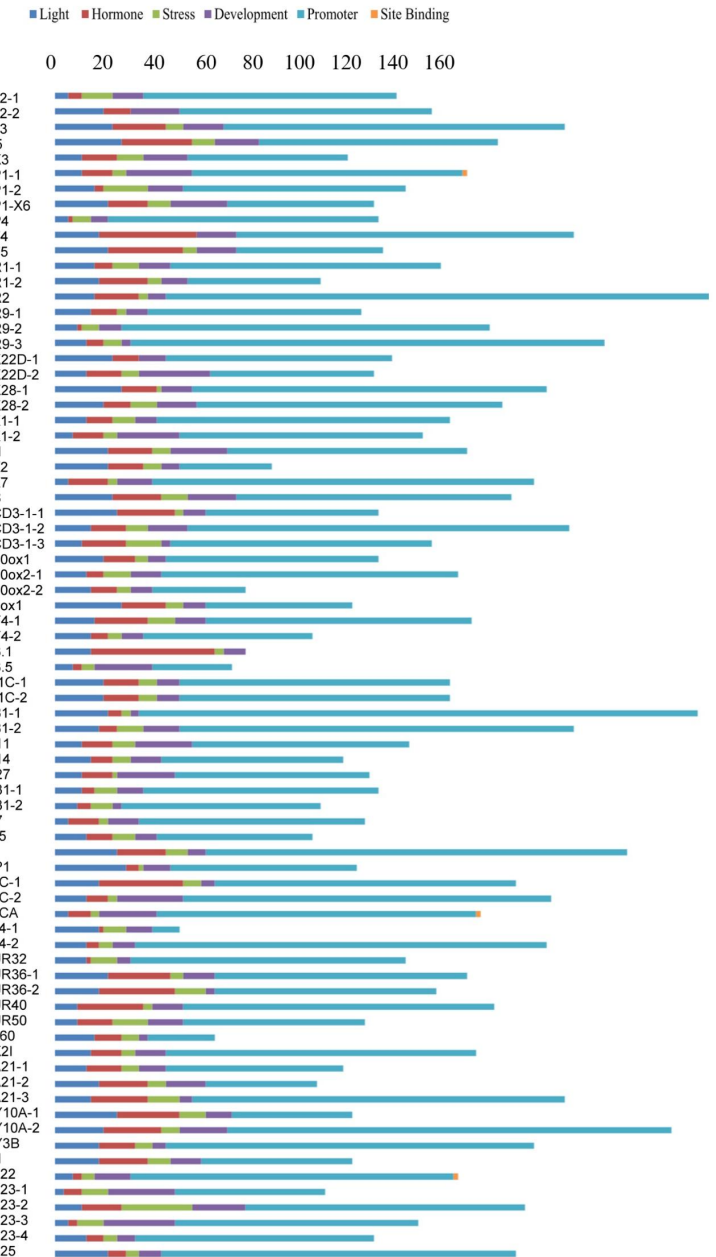

Supplement: Supplementary file 1 [file ijms-26-01102-s001.zip › ijms-3385732-supplementary.pdf]
